# Supporting the differential diagnosis of connective tissue diseases with neurological involvement by blood and cerebrospinal fluid flow cytometry

**DOI:** 10.1186/s12974-023-02733-w

**Published:** 2023-02-23

**Authors:** Michael Heming, Louisa Müller-Miny, Leoni Rolfes, Andreas Schulte-Mecklenbeck, Tobias J. Brix, Julian Varghese, Marc Pawlitzki, Hermann Pavenstädt, Martin A. Kriegel, Catharina C. Gross, Heinz Wiendl, Gerd Meyer zu Hörste

**Affiliations:** 1 Department of Neurology With Institute of Translational Neurology, University Hospital Münster, Albert-Schweitzer-Campus 1, Building A1, 48149 Münster, Germany; 2 Department of Neurology, University Hospital Düsseldorf, Düsseldorf, Germany; 3 Institute of Medical Informatics, University of Münster, Münster, Germany; 4 Division of General Internal Medicine, Nephrology and Rheumatology, Department of Medicine D, University Hospital of Münster, Münster, Germany; 5 Department of Translational Rheumatology and Immunology, Institute of Musculoskeletal Medicine, University of Münster, Münster, Germany; 6 Section of Rheumatology and Clinical Immunology, Department of Medicine, University Hospital Münster, Münster, Germany

**Keywords:** Cerebrospinal fluid, Connective tissue disease, Systemic lupus erythematosus, Flow cytometry

## Abstract

**Objective:**

Neurological manifestations of autoimmune connective tissue diseases (CTD) are poorly understood and difficult to diagnose. We here aimed to address this shortcoming by studying immune cell compositions in CTD patients with and without neurological manifestation.

**Methods:**

Using flow cytometry, we retrospectively investigated paired cerebrospinal fluid (CSF) and blood samples of 28 CTD patients without neurological manifestation, 38 CTD patients with neurological manifestation (N-CTD), 38 non-inflammatory controls, and 38 multiple sclerosis (MS) patients, a paradigmatic primary neuroinflammatory disease.

**Results:**

We detected an expansion of plasma cells in the blood of both N-CTD and CTD compared to non-inflammatory controls and MS. Blood plasma cells alone distinguished the clinically similar entities N-CTD and MS with high discriminatory performance (AUC: 0.81). Classical blood monocytes indicated higher disease activity in systemic lupus erythematosus (SLE) patients. Surprisingly, immune cells in the CSF did not differ significantly between N-CTD and CTD, while CD4^+^ T cells and the CD4^+^/CD8^+^ ratio were elevated in the blood of N-CTD compared to CTD. Several B cell-associated parameters partially overlapped in the CSF in MS and N-CTD. We built a machine learning model that distinguished N-CTD from MS with high discriminatory power using either blood or CSF.

**Conclusion:**

We here find that blood flow cytometry alone surprisingly suffices to distinguish CTD with neurological manifestations from clinically similar entities, suggesting that a rapid blood test could support clinicians in the differential diagnosis of N-CTD.

**Supplementary Information:**

The online version contains supplementary material available at 10.1186/s12974-023-02733-w.

## Background

Systemic lupus erythematosus (SLE), granulomatosis with polyangiitis (GPA), and primary Sjögren's syndrome (SS) are three of the most common autoimmune connective tissue diseases and vasculitides, which we will refer to as connective tissue diseases (CTDs) for simplicity. Although their exact triggers are still under debate, both genetic and microbiota/environmental factors are likely to contribute [1].

Neurological manifestations can occur in CTD patients and generally indicate tissue inflammation and an unfavorable prognosis [2, 3]. Neurological sequelae are especially common in SLE, but their prevalence is highly variable, ranging between 20 and 50%, and include seizures, cognitive dysfunction, headaches, stroke, psychosis, and aseptic meningitis [4]. Neurological involvement in SS is also common, and peripheral neuropathy is likely its most common complication (2–10%) [5]. GPA patients develop neurological manifestations in ~ 30%, especially peripheral and cranial neuropathy [6].

Differentiating CTD without neurological manifestation from CTD patients with neurological manifestations (hereafter referred to as N-CTDs) is often challenging but important to guide treatment decisions because patients with N-CTD have increased mortality [7] and lower quality of life [8]. Additionally, patients with N-CTD often require stronger immunosuppressive treatments, such as cyclophosphamide, compared to those with CTD [9]. Early diagnosis of N-CTDs can improve treatment responses [10]. MRI scans, blood samples, electrophysiology, and EEGs often do not show abnormalities, and if they do, they are not specific for N-CTD. Demyelinating syndrome, transverse myelitis, and vasculitis [11] can be key features of N-CTDs, such as neuropsychiatric lupus erythematosus [9], which are often indistinguishable from MS lesions on MRI [12]. In fact, several studies in the field of neuroradiology [13–16] show that in a clinical context it is often difficult to differentiate between these two diseases based on MRI data alone. Additionally, N-CTDs can mimic MS and in the current diagnostic criteria for MS it is mandatory to rule out differential diagnoses, including SLE and SS [17]. Better diagnostic and prognostic surrogates for N-CTD are therefore a major unmet medical need.

While the systemic pathogenesis of CTDs has been extensively studied [18], neurological involvement in CTDs is still poorly understood. In neuropsychiatric lupus, the cerebrospinal fluid (CSF) shows elevated levels of cytokines that promote B cell activation and differentiation (APRIL, BAFF) [19] as well as intrathecal immunoglobulin synthesis [20]. Accordingly, several autoantibodies (anti-NMDAR, anti-ribosomal P, anti-phospholipid) were described in SLE with neurological manifestations [21]. However, the diagnostic value of those antibodies is limited [22]. Collectively, previous findings suggest an important role for the B cell lineage in the CSF of SLE patients with neurological manifestation. The exact pattern of CSF immunity in other N-CTD remains unknown.

Flow cytometry analysis of CSF leukocyte composition is a powerful tool for diagnosing and understanding brain immunity [23–26]. CSF cytokine and antibody analyses [19] have been useful in improving understanding of neurological manifestations in CTD, but immune cell profiling of CSF cells from CTD patients has not yet been reported. Therefore, we conducted a comprehensive retrospective flow cytometry analysis of blood and CSF cells from CTD and N-CTD patients.

## Methods

### Patient cohorts

We screened patients who were admitted to the University Hospital Münster between 2011 and 2020 and received a diagnostic lumbar puncture, including flow cytometry, for the ICD-10 diagnoses M32 (systemic lupus erythematosus), M35.0 (Sjögren syndrome), and M31.3 (granulomatosis with polyangiitis). Flow cytometry of the CSF and blood is routinely performed in our center. CTD patients were selected according to the ACR/EULAR criteria. SLE patients fulfilled the 2019 ACR/EULAR criteria for SLE [27], SS patients the 2016 ACR/EULAR criteria for SS [28], and GPA patients the 2022 ACR/EULAR criteria for GPA [29]. Neurological manifestation (N-CTD) was defined as neurological signs or symptoms attributed to the primary disease. We queried our local database for patients with multiple sclerosis and somatoform disorders who underwent a lumbar puncture with a flow cytometric assessment. MS patients fulfilled the 2017 revised McDonald criteria [17], were first diagnosed at the time of sampling, and were treatment-naive.

### Flow cytometry and routine CSF analysis

LPs were performed under sterile conditions, and CSF was promptly analyzed. CSF was centrifuged at 300*g* for 15 min, the supernatant was removed, and CSF cells were stained with different antibodies as described previously [23–25]. Briefly, we performed flow cytometry on a Navios flow cytometer (Beckman Coulter). Cells were incubated in VersaLyse buffer and stained with anti-human antibodies (Beckman Coulter). The clone names are provided in parentheses: CD3 (UCHT1); CD4 (13B8.2); CD8 (B9.11); CD14 (RMO52); CD16 (3G8); CD19 (J3–119); CD45 (J.33); CD56 (C218); CD138 (B-A38); and HLA-DR (Immu-357). The gating scheme is depicted in Additional file 1: Fig. S1. Cell population size was defined as the number of gated cell events relative to the events of the corresponding parent gate (Additional file 1: Fig. S1). CSF cells were counted with a Fuchs-Rosenthal chamber. Protein concentration, IgA, IgG, and IgM levels were studied by nephelometry (BN ProSpec^®^, Siemens). A Reiber scheme was created and evaluated for the presence of a blood–CSF barrier disruption (BCBD). Oligoclonal bands (OCBs) were detected via isoelectric focusing followed by silver nitrate staining. In detail, a mixture of 5 µl serum and 100 µl cell-free CSF was diluted to a concentration of 25 mg/l IgG using IgG Sample Diluent (Serva). 20 µl of the diluted sample was then added to the wells of a FocusGel 6–11 24S (Serva) on a Multiphor II cooling plate (Pharmacia Biotech). The mixture was then electrophoresed. Next, the gel was transferred to a processor and gel stainer (GE Healthcare), fixed, and covered. The staining process is automated, and upon completion, the gel was dried at room temperature and sealed with Mylar conservation sheets (GE Healthcare).

### Statistics

The computational analysis was carried out with *R* 4.1.1. The Mann–Whitney *U* test was used when comparing two groups and the Kruskal–Wallis test with the Dunn test as a post hoc test when comparing multiple groups. The Benjamini–Hochberg method was used for multiple testing corrections. The significance level was set at 0.05. We used propensity scores estimated by logistic regression and nearest neighbor matching with the *MatchIt* R package [30] to identify sex- and age-matched somatoform and MS patients in our database. We adjusted for the therapy with the *datawizard* R package. Briefly, a linear model was fitted for each variable with the therapy status as a predictor. The residuals were computed and used for further analysis. The box plots, the variable importance plot, and the ROC curves were generated with the *ggplot2 R* package. The correlation plot was produced with the R package *corrplot* based on the Spearman coefficient. The data were clustered hierarchically with complete linkage and the Euclidean distance measure. The heatmaps were created with the *R* package *pheatmap*. In a first step, Yeo-Johnson transformation was performed, followed by centering and scaling with the *recipes R* package. Next, the mean of each parameter for each cohort was calculated and visualized in a heatmap. Rows were clustered hierarchically with complete linkage and the Euclidean distance measure. We performed receiver operating characteristic (ROC) analysis with the R package *pROC* [31]. A ROC analysis permits a systematic evaluation of the sensitivity and specificity of a test represented by the area under the curve (AUC) values, which range from 0.5 (uninformative) to 1 (perfect) [32]. The machine learning algorithms were evaluated with the *tidymodels R* package. First, the data were split into training (75%) and test (25%) data stratified according to the outcome. The data were then preprocessed by removing sparse variables and performing a Yeo-Johnson transformation, centering, and scaling. Lasso regression was performed with the *glmnet* package [33]. We trained the model with repeated tenfold cross-validation with 100 repeats to take the small number of samples into account. The best model was chosen based on the highest AUROC. The resulting variable importance scores were determined with the *R* package *vip*.

## Results

### Patient cohort and interdependency of individual flow cytometry parameters

**Fig. 1:**
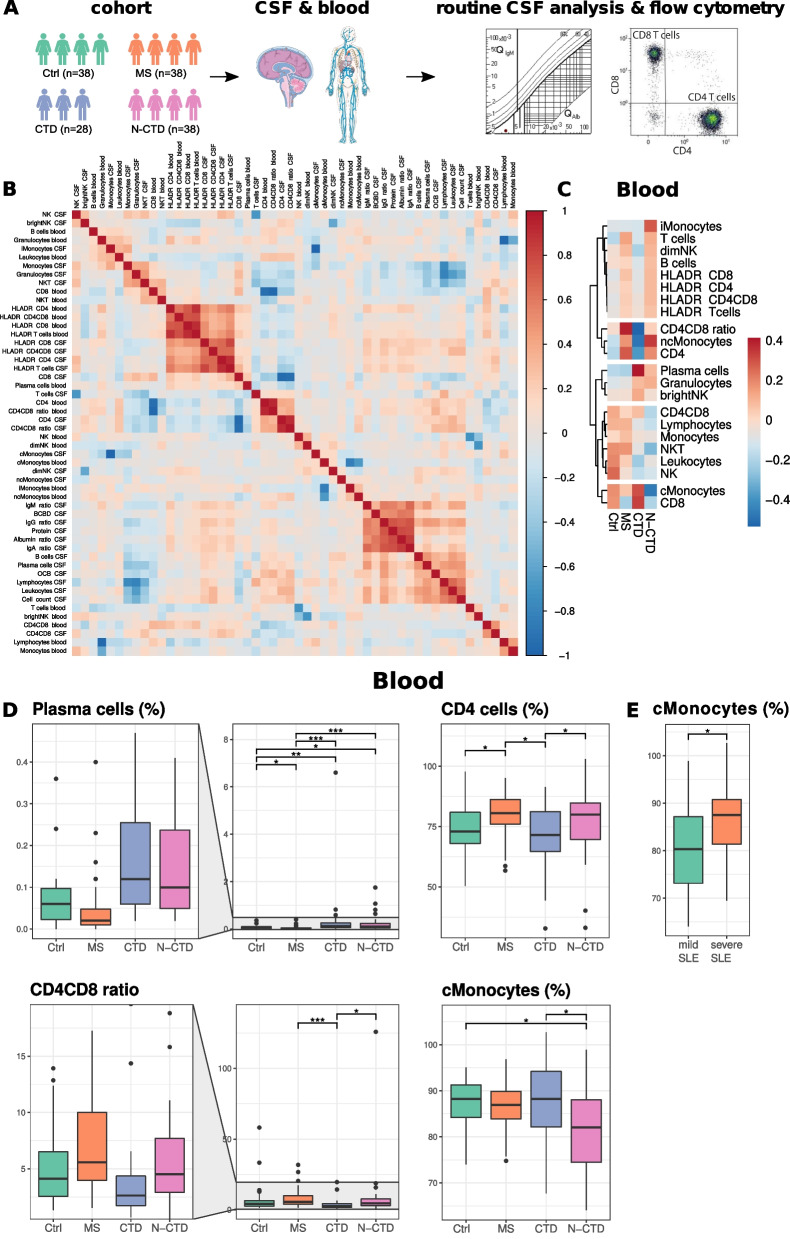
Elevated blood plasma cells in CTD and N-CTD. **A** Scheme illustrating the study design––Cerebrospinal fluid (CSF) and blood from 38 somatoform control patients (Ctrl), and 38 patients with MS (multiple sclerosis), 28 CTD (connective tissue disease without neurological manifestation), and 38 N-CTD (connective tissue disease with neurological manifestation)––was analyzed by flow cytometry using a predefined antibody panel (Methods), and additional standard CSF analysis was performed as part of the diagnostic workup. Data were queried and analyzed retrospectively. **B** Spearman correlation between all analyzed parameters. Data were clustered hierarchically. **C** Heatmap of the group means of the blood parameters. The group mean of each blood parameter was determined, then normalized, and clustered hierarchically (see "Methods" section). **D** Blood parameters that showed significant alterations in any comparison are visualized in box plots categorized by disease group. Immune cell frequencies are shown as percentages of their parent gate (Methods). The boxes display the lower quartile, median, and upper quartile and the whisker includes 1.5 times the interquartile range. The statistical significance was calculated with the Kruskal–Wallis test with post hoc Dunn’s test. The *p*-values were adjusted with the Benjamini-Hochberg’s method. **E** SLE patients were categorized into “severe” or “mild” based on their ACR/EULAR score (threshold 20) [3] and visualized in a box plot similar to **D**. The statistical significance was determined with the Mann–Whitney U test. *p < 0.05, **p < 0.01 ***p < 0.001. Parts of **A** have been adapted from Servier Medical Art

Whether N-CTDs display immune cell alterations distinct from CTDs without neurological symptoms and other neuroinflammatory diseases remains unknown. We therefore retrospectively analyzed available flow cytometry data of paired blood and CSF samples and routine CSF parameters of 28 CTD and 38 N-CTD patients, who had been admitted to our center during the past 10 years and underwent a lumbar puncture as part of the diagnostic workup (Fig. 1A). Notably, all CSF samples collected during regular working hours at our center are analyzed using a standardized flow cytometry panel covering most leukocyte lineages (Methods). A detailed clinical characterization of the CTDs, including organ manifestations, antibody status, ACR/EULAR scores, and neurological signs and symptoms of each patient, was extracted from the electronic health records (Additional file 2: Table S1). The majority of patients were diagnosed with SLE (46 of 66), a smaller proportion with GPA or SS (Table 1). We added data from propensity score-matched somatoform control patients (Ctrl) and therapy-naive relapsing–remitting MS patients, a paradigmatic CNS autoimmune disease (Methods). Consequently, the age and sex ratios were comparable across all groups (Table 1).

**Table 1:** Characteristics of study patients

	No.	Median age	% Female	GPA	SLE		SS
Ctrl	38	46.4	76.3	0	0		0
MS	38	44.4	81.6	0	0		0
CTD	28	50.1	67.9	6	17		5
N-CTD	38	46.6	76.3	4	29		5

We then tested for the interdependence of the available blood and CSF parameters and identified several modules of correlating parameters (Fig. 1B). Blood and CSF T cell populations expressing the activation marker HLA-DR (e.g., “HLADR T cells blood” and “HLADR T cells CSF”) formed one large correlating cluster (Fig. 1B). T cell activation is thus shared across compartments. A second module included CSF immunoglobulin (Ig) ratios, blood–CSF barrier disruption (BCBD), CSF protein, albumin ratio, CSF B cells, CSF plasma cells, and oligoclonal bands (OCB). Intrathecal B cell expansion and immunoglobulin synthesis are thus reflected in a set of co-regulated parameters.

### Blood plasma cells were increased in CTD irrespective of neurological involvement.

We first aimed to understand how connective tissue diseases affected the immune cell composition in the blood. Blood plasma cells were significantly elevated in both CTD (0.41%) and N-CTD (0.22%) compared to Ctrl (0.07%) and MS (0.05%) patients (Fig. 1C, D). MS is known to induce gross compositional changes in CSF but not in blood leukocytes [34]. In contrast to the brain autoimmune disease MS, blood plasma cell proportions are thus increased in systemic autoimmune CTD and may help diagnose CTD, irrespective of neurological involvement. We next sought to identify blood parameters that distinguished N-CTD from CTD patients. We found that in the blood CD4^+^ T cells and the CD4^+^/CD8^+^ ratio were increased in N-CTD compared to CTD (CD4: 77.7% vs. 71.7%; CD4^+^/CD8^+^ ratio: 8.75 vs. 3.97), while classical monocytes were decreased in N-CTD (81.8% vs 87.4%) (Fig. 1C, D). Neurological involvement of systemic autoimmunity is thus associated with specific alterations of innate and adaptive immune cells in the blood. This indicates diagnostic potential of blood parameters.

### Classical monocytes in the blood are associated with higher SLE activity

Disease severity in SLE shows high inter-individual variability. SLE patients with an ACR/EULAR score of ≥ 20 have higher disease activity, less remission, and more often require immunosuppressive therapy [3]. We aimed to understand if CSF or blood parameters differ between SLE patients with high and low disease activity. Therefore, we dichotomized SLE patients into mild (*n* = 21) and severe (*n* = 25) severity based on their ACR/EULAR scores as described in the literature [27]. In our cohort, severe cases were—unexpectedly—more prevalent in CTD than in N-CTD (CTD: 3 mild, 14 severe; N-CTD: 18 mild, 11 severe). This distribution is most likely due to recruitment bias. All of the patients were recruited at a tertiary referral hospital, which preferentially treats patients with severe SLE. Despite these limitations, severely affected SLE patients showed an increase in classical monocytes in the blood (86.7% vs. 79.8%) (Fig. 1E). Classical monocytes remained significantly higher in severe SLE patients after adjusting for imbalances in the distribution of CTD and N-CTD patients within the severity groups by multiple regression analysis (*p* = 0.03). All other blood and CSF parameters did not differ significantly between severe and mild SLE patients. The group size of the other CTDs was insufficient to subset based on severity. Collectively, this provides evidence that the decrease in classical monocytes in N-CTD is not disease specific but rather reflects disease severity. In contrast, the increase in CD4^+^ T cells and the CD4^+^/CD8^+^ ratio in the blood likely represent “neurologic-specific” surrogates of CTD.

### N-CTD shares some, but not all features with MS in the CSF

**Fig. 2:**
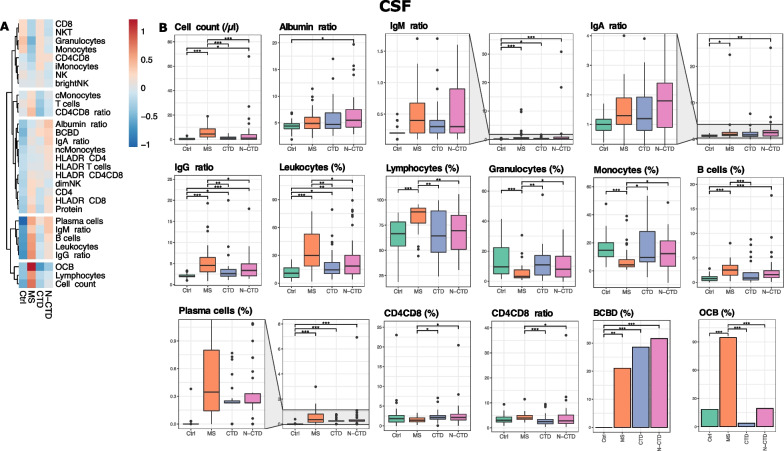
Partial overlap of the CSF immune profile of N-CTD and MS. **A** Heatmap of the group means of the CSF parameters. The group mean of each CSF parameter was calculated, normalized and clustered hierarchically (see “Methods” section). **B** CSF parameters that showed significant changes in any comparison between the diseases are shown in bar plots and box plots, categorized by disease group. Immune cell frequencies are displayed as percentages of their parent gate (Methods). The boxes display the lower quartile, median, and upper quartile and the whisker includes 1.5 times the interquartile range. The statistical significance was calculated with the Kruskal–Wallis test with post hoc Dunn’s test. The p-values were adjusted with the Benjamini-Hochberg’s method. *p < 0.05, **p < 0.01 ***p < 0.001

We next investigated whether analysis of the CSF, which ensheaths the brain, could shed more light on neurology-specific mechanisms in CTDs. However, no single CSF parameter differed significantly between N-CTD and CTD (Fig. 2A, B). In stark contrast to the primary brain autoimmune disease MS, laboratory surrogates distinguishing N-CTD from CTD are thus more readily detectable in the blood (Fig. 1D) than in CSF (Fig. 2B). One may thus speculate that peripheral—not intrathecal—mechanisms dominantly determine CNS manifestations of CTD.

Our next goal was to find markers that would aid in the differential diagnosis between MS and N-CTD because MRI and clinical manifestations of N-CTD can be indistinguishable from MS in some patients [12]. We replicated the increase in lymphocytes, elevated B cells, plasma cells, and intrathecal immunoglobulin (Ig) synthesis known to characterize the CSF of MS patients. In addition, a BCBD was observed in > 20% and oligoclonal bands (OCBs) in over 90% of MS patients (Fig. 2A, B). Interestingly, N-CTD patients shared several features of the MS CSF immune profile, including intrathecal IgG, IgA and IgM synthesis, a BCBD, and increased B and plasma cells compared to Ctrl patients (Fig. 2A, B). This indicates intrathecal B cell expansion. In contrast, most of those changes were not observed in CTD compared to Ctrl patients (Fig. 2A, B). However, compared to MS, N-CTD patients showed fewer OCBs (19.4% vs. 94.6%) and a reduced IgG CSF/blood ratio (3.97 vs. 5.57). Moreover, leukocytes, lymphocytes, and the CD4^+^/CD8^+^ ratio were reduced, while granulocytes, monocytes, and double-positive T cells were elevated in N-CTD compared to MS patients (Fig. 2A, B). In summary, we identified a partially distinct immune cell profile that distinguishes N-CTD from MS.

All MS samples had been collected in relapse, while several CTD (23/28) and N-CTD (9/38) patients were in remission at the time of CSF collection. We wondered whether this difference introduced a relevant bias in our results. Therefore, we subdivided CTD and N-CTD patients into patients in remission and relapse (Additional file 1: Fig. S2). However, we did not find significant differences between relapse and remission in the blood or CSF when subsetting N-CTD and CTD patients for relapse/remission at sampling (Additional file 1: Fig. S2). Additionally, MS patients were sampled at the time of diagnosis, whereas disease duration varied widely in CTD and N-CTD patients. The CSF CD4^+^/CD8^+^ ratio negatively correlated with disease duration, while the remaining CSF and blood parameters with significant differences between the diseases did not correlate significantly with the disease duration (Additional file 1: Fig. S3). This indicates that the observed reduction of the CD4^+^/CD8^+^ ratio in the CSF in N-CTD compared to MS patients could be explained by longer disease duration. In contrast, this suggests that the other disease-related changes we observed were not caused by relevant temporal sampling bias.

### Blood plasma cells differentiate N-CTD from MS

**Fig. 3:**
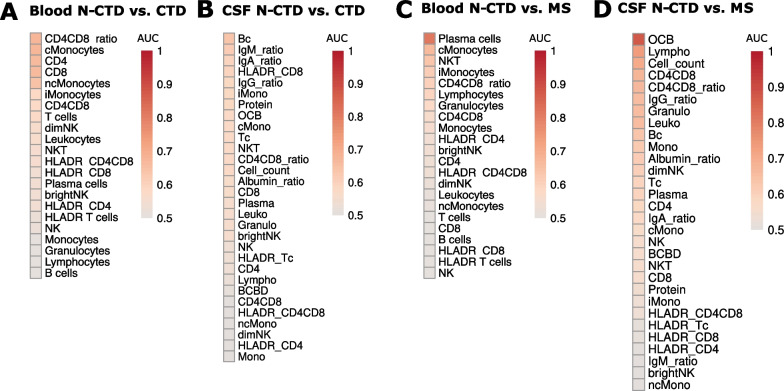
Blood plasma cells can distinguish N-CTD from CTD. **A**–**D** The AUC (area under the curve) values of the ROC analysis of N-CTD vs. CTD in the blood (**A**) and the CSF (**C**), and of N-CTD vs. MS in the blood (**B**) and the CSF (**D**) are visualized in a heatmap sorted by AUC value. Possible AUC values range from 0.5 (uninformative) to 1 (perfect) and measure the discriminatory ability (combined specificity and sensitivity) of the parameter

We next aimed to quantify the diagnostic power of the parameters we had identified. We systematically quantified the discriminative power of blood and CSF separately. We focused on the clinically relevant comparisons between N-CTD vs. MS and N-CTD vs. CTD. In blood, the CD4^+^/CD8^+^ T cell ratio (AUC 0.68) was the best parameter to distinguish N-CTD from CTD patients (Fig. 3A, Additional file 3: Table S2). Interestingly, CSF parameters performed slightly worse in differentiating N-CTD from CTD (B cells: AUC 0.63) (Fig. 3B, Additional file 3: Table S2). In line with our findings in Fig. 1, the best parameter to differentiate N-CTD from MS patients in the blood was plasma cells (AUC 0.81), which were increased in N-CTD compared to MS (Figs. 1D, 3C, Additional file 3: Table S2). This is consistent with our previous observation that N-CTD alters the peripheral blood more than MS. The two best CSF parameters for distinguishing N-CTD from MS patients were OCB (AUC 0.88) and lymphocytes (0.74), which were elevated in MS compared to N-CTD (Figs. 2B, 3D, Additional file 3: Table S2). This provides evidence that in addition to OCB, which requires a lumbar puncture, blood plasma cells can already assist clinicians in the challenging differential diagnosis of N-CTD vs. MS.

### N-CTD can be differentiated from MS with high discriminatory power with multivariable models of blood and CSF immune cells

**Fig. 4:**
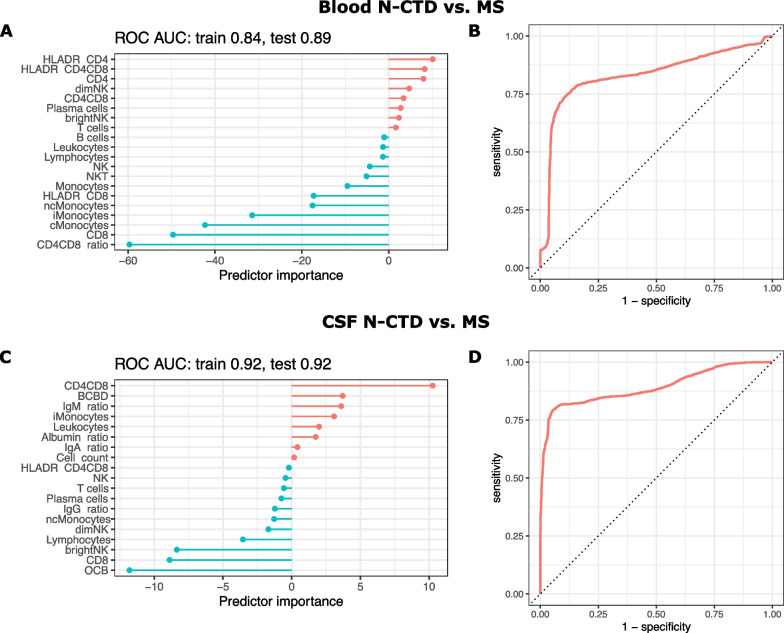
Multivariable models of immune cells differentiate N-CTD from MS with high discriminatory capacity. **A–D** Lasso regression was fitted on the blood (**A**, **B**) and CSF (**C**, **D**) parameters to classify N-CTD and MS. Predictors with positive predictor importance values (**A**, **C**) are colored in red. High values of these parameters increase the probability of N-CTD. Predictors with negative values are colored in blue and high values increase the probability of MS

To capitalize on the many variables collected by flow cytometry, we tested the discriminative power of variable combinations compared to single variables. Therefore, we evaluated several machine learning models (logistic regression, lasso and ridge logistic regression, support vector machines, bagged trees, random forest) with the AUC of the ROC analysis as a performance measure. When differentiating between N-CTD and MS, the lasso regression approach showed the best performance. Additionally, lasso regression inherently removes unimportant features, thus making the model easier to use and interpret. In blood, the resulting model had a high AUC in both the training (0.84) and test (0.89) datasets (Fig. 4A, B). The most important variables in this model were CD4^+^/CD8^+^ ratio and CD8^+^ T cells, followed by monocyte subpopulations (Fig. 4A). Both variables increased the probability of N-CTD. In CSF, the best model to distinguish N-CTD from MS showed slightly better performance (AUC 0.92 in the train and test data). High values of OCB, CD8+ T cells, and bright NK cells elevated the probability of MS, while high counts of double-positive CD4^+^CD8^+^ T cells increased the probability of N-CTD in this model. Combining both blood and CSF parameters resulted in comparable performance of the model (AUC train: 0.92; AUC test: 0.9). In contrast, when distinguishing N-CTD from CTD, the multivariable lasso regression performed similarly to the single variables (Additional file 1: Fig. S4). Collectively, combinations of either blood or CSF parameters can equally support the differential diagnosis of N-CTD vs. MS with high accuracy. Flow cytometry of the blood could thus allow a rapid and less-invasive procedure to aid clinicians’ diagnostic accuracy in these clinically similar entities.

## Discussion

The pathogenesis of CTD and its neurological manifestations is poorly understood and the differentiation from other neuroinflammatory diseases remains challenging. Capitalizing on existing multidimensional flow cytometry data, we identified an expansion of plasma cells in the blood in both N-CTD and CTD compared to non-inflammatory controls and MS. The differential diagnosis between N-CTD and MS can be challenging [12]. Importantly, blood plasma cells also performed well in separating N-CTD and MS (AUC: 0.81). Classical monocytes in the blood were increased in severe SLE. While there were no significant differences between N-CTD and CTD in the CSF, the CD4^+^/CD8^+^ ratio and CD4^+^ T cells were increased in the blood of N-CTD. It is therefore tempting to speculate that peripheral—not intrathecal—immune alterations predominantly determine CNS manifestation in CTD. Moreover, we revealed that the CSF immune profile of N-CTD—but not that of CTD—was related to the primary brain autoimmune disease MS (CSF: cell count, leukocytes, B cells, plasma cells), but also notably distinct (e.g., OCB, IgG ratio, blood plasma cells, CSF double-positive T cells). We were able to construct a machine learning model that differentiated N-CTD from MS with high accuracy using either blood or CSF. Rapid flow cytometric analysis of the blood could potentially assist clinicians in differentiating N-CTD from clinically similar diseases.

Many of our observations were consistent with previous observations. We found a BCBD in CTD and N-CTD patients, as previously described in neuropsychiatric SLE [35]. Altered monocyte levels and their increased proinflammatory activity have been reported in various CTDs, but the abundance of monocyte subsets remains controversial [36, 37]. Patrolling monocytes promote kidney disease in SLE [38], and their impaired clearance of dying cells is an important concept in SLE pathogenesis [39]. Our findings suggest that classical monocytes can distinguish between severe and mild SLE.

Autoreactive B cells, plasma cells, and the resulting antibodies are known to be one of the main drivers of most CTDs [40, 41]. Blood plasma cells are increased in CTD and N-CTD patients and correlate with disease activity [42, 43]. We now observed a B cell-driven profile in the CSF of N-CTD patients with increased B cells, plasma cells, and intrathecal IgG, IgA, and IgM synthesis, which partially overlapped with the well-established CSF immune profile in MS [44, 45]. Accordingly, others detected intrathecal Ig synthesis and tertiary lymphoid structures in the choroid plexus in neuropsychiatric SLE patients [20, 46]. These plasma cells could locally produce autoantibodies in the CSF that drive neurological manifestations in CTD patients. Consequently, this also supports the use of B cell-directed therapies, such as belimumab for neurological manifestations of CTDs, which is successfully used as an add-on therapy for renal and extrarenal SLE [46].

Our study is limited by its retrospective design; associated confounding factors, especially treatment; a biased flow cytometry panel; a selection bias, as all patients were enrolled in a tertiary referral center and received a lumbar puncture; and a relatively small sample size. However, we adjusted for treatment with a regression model. We also found no evidence that inhomogeneities in disease status and progression biased our main results. Further prospective multicenter studies with larger cohorts are needed to validate our findings.

Collectively, we here provide a comprehensive CSF and blood analysis of the immune cell composition of patients with connective tissue diseases with and without neurological manifestations. Our analysis adds to the understanding of discrepancies and similarities between the immune profiles of N-CTD and CTD in CSF and blood. Moreover, we provide evidence that flow cytometric analysis of peripheral blood can support the clinically challenging but important differentiation between N-CTD and MS. Mechanistic understanding of diverse immune compartments can thus supplement the armamentarium in neuroimmunological diseases that are difficult to diagnose.

## Supplementary Information

**Additional file 1: Figure S1.** Gating scheme. **Figure S2.** Comparison of blood and CSF parameters between CTD and N-CTD in relapse and remission. **Figure S3.** Correlation analysis between disease duration and blood and CSF parameters in CTD and N-CTD. **Figure S4.** Multivariable models differentiating N-CTD from CTD.

**Additional file 2: Table S1.** Patient characteristics table.

**Additional file 3: Table S2.** ROC AUC values corresponding to Fig. 3.

## Data Availability

Data are available from the corresponding author upon reasonable request.

## Abbreviations

AUC: Area under the curve
BCBD: Blood–CSF barrier disruption
brightNK: CD56^bright^ natural killer cells
CTD: Connective tissue disease
CD4: CD4^+^ T cells
CD8: CD8^+^ T cells
CD4CD8: CD4^+^CD8^+^ T cells
CD4CD8_ratio: CD4^+^/CD8^+^ T cell ratio
CSF: Cerebrospinal fluid
cMonocytes: Classical monocytes
dimNK: CD56^dim^ natural killer cells
GPA: Granulomatosis with polyangiitis
Ig: Immunoglobulin
iMonocytes: Intermediate monocytes
MS: Multiple sclerosis
nMonocytes: Non-classical monocytes
NKT: Natural killer T cells
OCB: Oligoclonal band
PCA: Principal component analysis
ROC: Receiver operating characteristic
SLE: Systemic lupus erythematosus
SS: Sjögren syndrome
