# Supplementary material for: Supporting the differential diagnosis of connective tissue diseases with neurological involvement by blood and cerebrospinal fluid flow cytometry
Source: J Neuroinflammation. 2023 Feb 23;20:46. doi: 10.1186/s12974-023-02733-w (PMC9951507; doi:10.1186/s12974-023-02733-w)
Supplement: Supplementary file 1 — Additional file 1: Figure S1. Gating scheme. Figure S2. Comparison of blood and CSF parameters between CTD and N-CTD in relapse and remission. Figure S3. Correlation analysis between disease duration and blood and CSF parameters in CTD and N-CTD. Figure S4. Multivariable models differentiating N-CTD from CTD. [file 12974_2023_2733_MOESM1_ESM.pdf]

Additional file 1: Supplementary Figures

Figure S1 Gating Scheme:

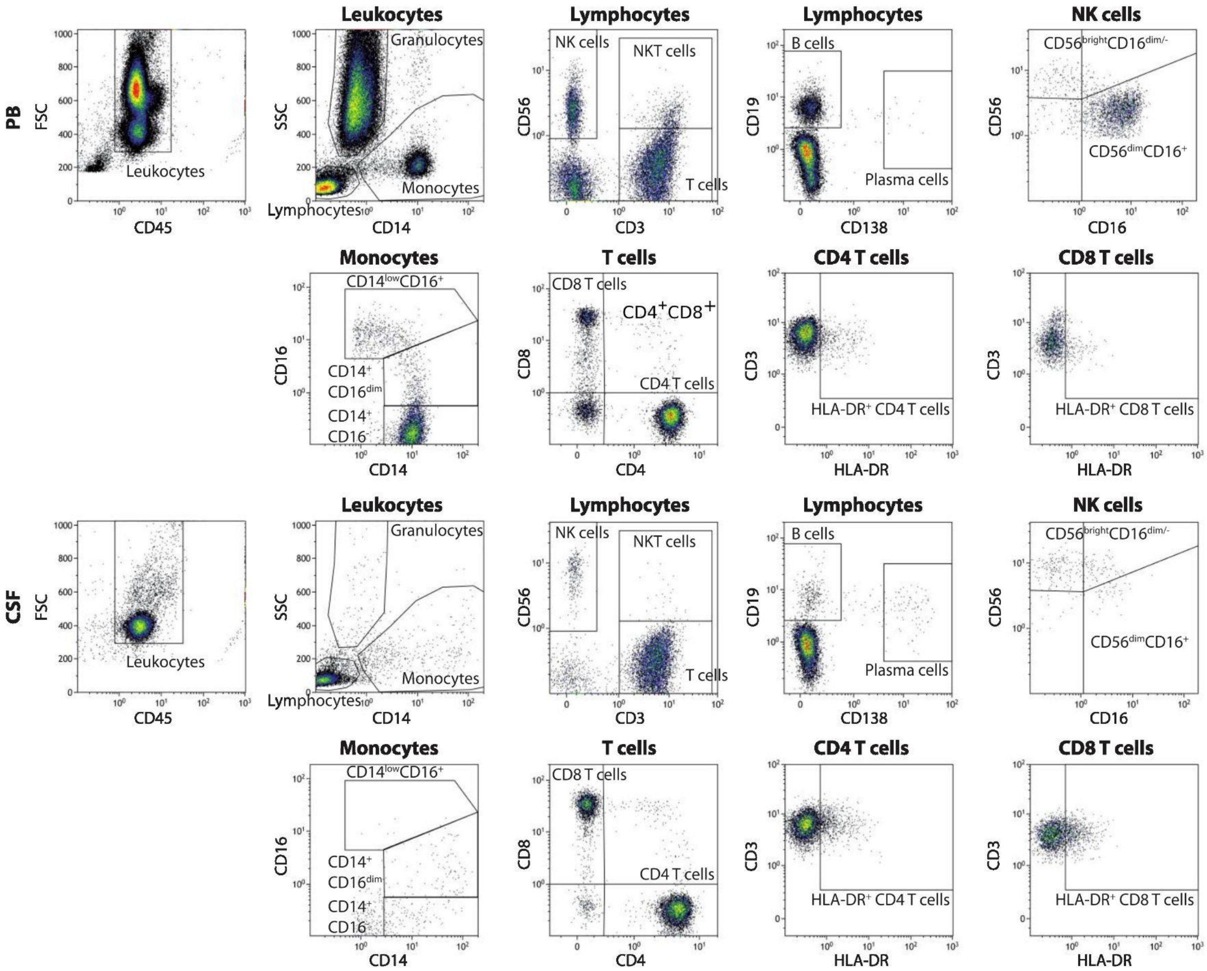

The flow cytometry gating process is shown in pseudocolor plots. Peripheral blood (PB) or cerebrospinal fluid (CSF) cells were gated on forward scatter (FSC), side scatter (SSC), followed by marker gating as shown.

Figure S2 Comparison of blood and CSF parameters between CTD and N-CTD in relapse and remission

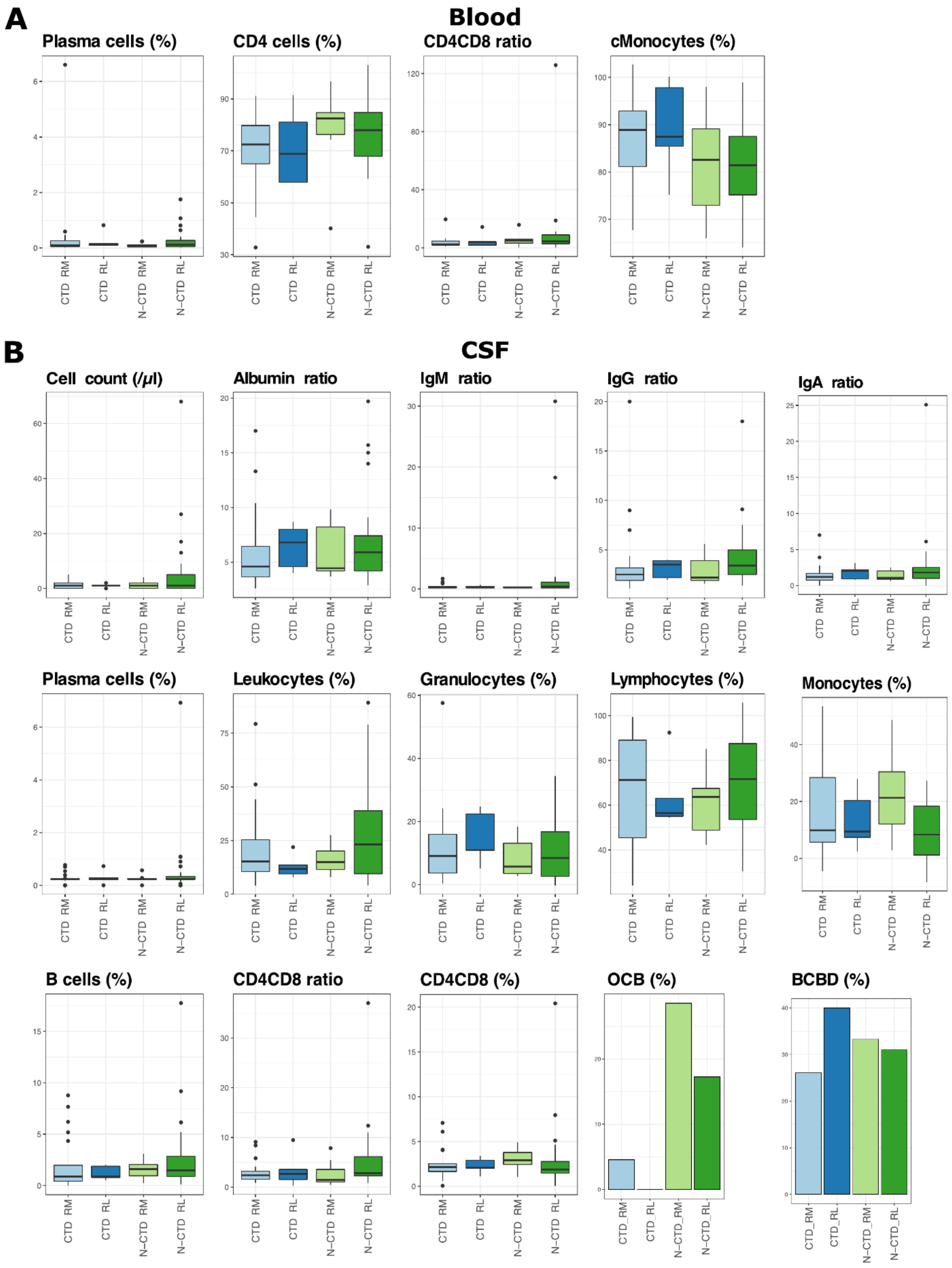

A-B CTD and N-CTD were subdivided into patients in remission (RM) and in relapse (RL). Blood (**A**) and CSF (**B**) parameters that showed significant differences between the diseases (Fig.s 1,2) are visualized in barplots and boxplots. Immune cell frequencies are displayed as percentages of their parent gate (Methods). The boxes display the lower quartile, median and upper quartile and the whisker includes 1.5 times the interquartile range. The statistical significance was calculated with the Kruskal-Wallis test with post-hoc Dunn's test. The p values were adjusted with the Benjamini-Hochberg's method.

Figure S3 Correlation analysis between disease duration and blood and CSF parameters in CTD and N-CTD

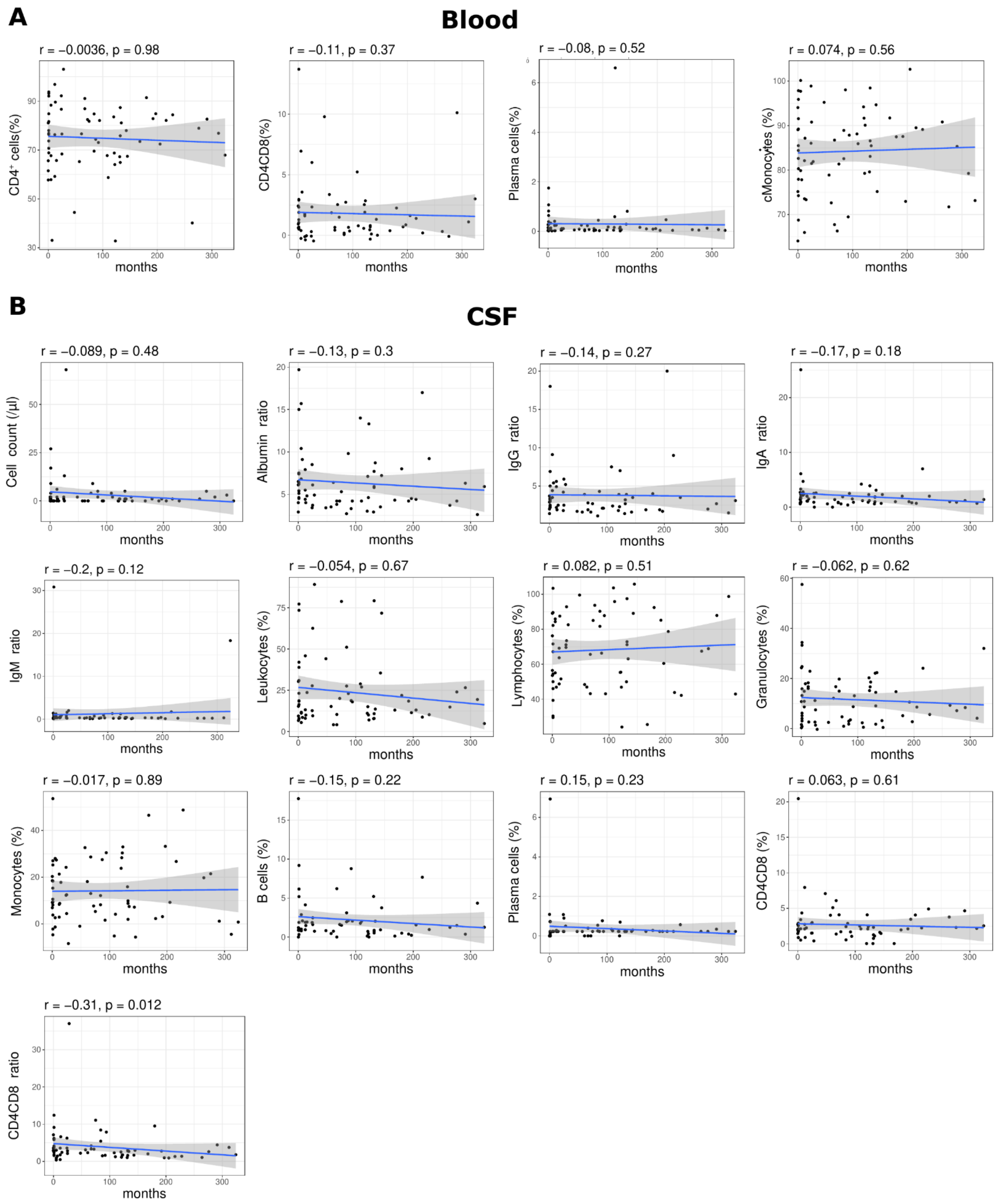

A-B Spearman correlation between the disease duration and blood (**A**) and CSF (**B**) parameters that showed significant differences between the diseases (Fig.s 1,2). The blue line displays the linear regression, the gray area visualizes the standard error.

Figure S4 Multivariable models differentiating N-CTD from CTD

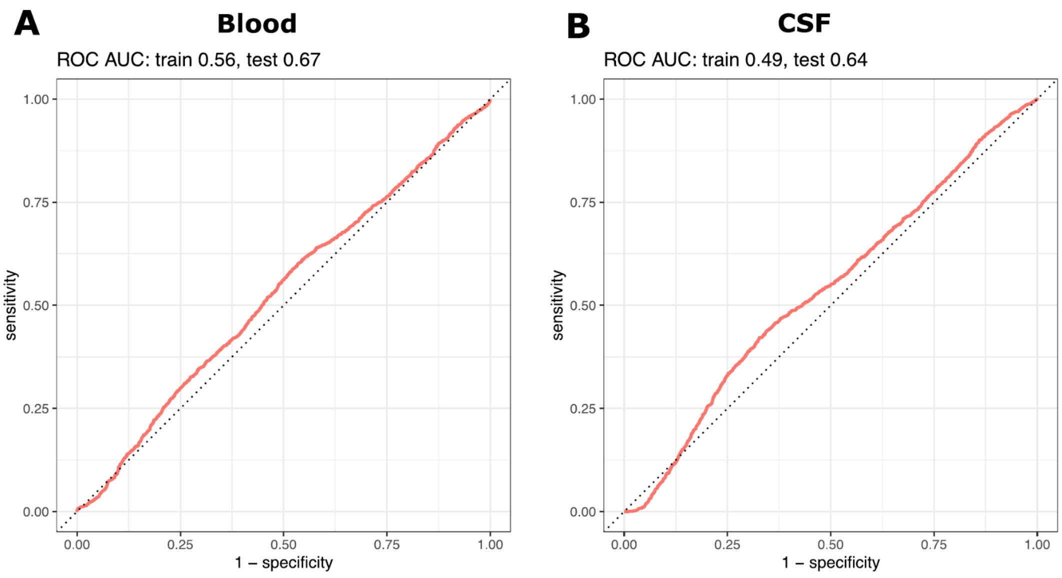

A-B To classify N-CTD and CSF, a Lasso regression was fitted on the blood (**A**) and CSF (**B**) variables.
